# Prevalence, drug resistance, and genotypic diversity of the RD^Rio^ subfamily of *Mycobacterium tuberculosis* in Ecuador: a retrospective analysis for years 2012–2016

**DOI:** 10.3389/fpubh.2024.1337357

**Published:** 2024-04-04

**Authors:** Bernardo Castro-Rodriguez, Greta Franco-Sotomayor, Jose Manuel Benitez-Medina, Greta Cardenas-Franco, Natalia Jiménez-Pizarro, Camilo Cardenas-Franco, Juan Luis Aguirre-Martinez, Solon Alberto Orlando, Javier Hermoso de Mendoza, Miguel Angel Garcia-Bereguiain

**Affiliations:** ^1^One Health Research Group, Universidad de las Américas, Quito, Ecuador; ^2^Instituto Nacional de Investigación en Salud Pública “Leopoldo Izquieta Pérez”, Guayaquil, Ecuador; ^3^Universidad Católica Santiago de Guayaquil, Guayaquil, Ecuador; ^4^Departamento de Sanidad Animal, Facultad de Veterinaria, Universidad de Extremadura, Cáceres, Spain; ^5^Universidad Espiritu Santo, Guayaquil, Ecuador

**Keywords:** *Mycobacterium tuberculosis*, RD^Rio^ subfamily, Spoligotyping, MIRU-VNTR, tuberculosis, Ecuador

## Abstract

**Introduction:**

A major sublineage within the *Mycobacterium tuberculosis* (MTB) LAM family characterized by a new in-frame fusion gene Rv3346c/55c was discovered in Rio de Janeiro (Brazil) in 2007, called RD^Rio^, associated to drug resistance. The few studies about prevalence of MTB RD^Rio^ strains in Latin America reported values ranging from 3% in Chile to 69.8% in Venezuela, although no information is available for countries like Ecuador.

**Methods:**

A total of 814 MTB isolates from years 2012 to 2016 were screened by multiplex PCR for RD^Rio^ identification, followed by 24-loci MIRU-VNTR and spoligotyping.

**Results:**

A total number of 17 MTB RD^Rio^ strains were identified, representing an overall prevalence of 2.09% among MTB strains in Ecuador. While 10.9% of the MTB isolates included in the study were multidrug resistance (MDR), 29.4% (5/17) of the RD^Rio^ strains were MDR.

**Discussion:**

This is the first report of the prevalence of MTB RD^Rio^ in Ecuador, where a strong association with MDR was found, but also a very low prevalence compared to other countries in Latin America. It is important to improve molecular epidemiology tools as a part of MTB surveillance programs in Latin America to track the transmission of potentially dangerous MTB stains associated to MDR TB like MTB RD^Rio^.

## Introduction

Tuberculosis (TB) is one of the leading causes of death from a single infectious agent, ranking just behind the coronavirus (COVID-19) and above HIV-AIDS (1). The disease is caused by members of the *Mycobacterium tuberculosis* Complex (MTBC) which are intracellular, rod-shaped, aerobic bacteria with a lipid-rich cell wall structured with mycolic acids that confer specific microbiological characteristics, like resistance to detergents, slow growth, and reaction to acid-fast stain methods. The main mechanism of spreading occurs by aerosols, affecting the lungs (pulmonary TB) and other organs (extrapulmonary TB) (1–3).

*Mycobacterium tuberculosis* comprehends a group of seven highly related lineages that infect mainly humans. Global distribution of these lineages differs greatly (4). In Latin America, the most frequent MTBC strains belong to the Euro-American lineage 4, which includes the Latin American-Mediterranean sublineage (LAM) (5–21). In Ecuador, there is a single report about population structure of MTBC showing that LAM sublineage is predominant (6). Moreover, LAM clonal complex comprised by Ecuadorian and Colombian strains were reported, suggesting transnational transmission of TB (6).

The genetic diversity in the MTBC species finds its origins in genomic alterations in targeted mycobacterial segments like region-of-difference (RD) loci that are used as specific markers for *M. tuberculosis* lineages (22, 23). In 2007, Lazzarini and collaborators applied a deletion-based PCR protocol to a collection of MTBC samples from Rio de Janeiro, Brazil, and identified a new in-frame fusion gene Rv3346c/55c that marks a major sublineage within the LAM family. This long sequence polymorphism results from a homologous recombination deletion between Rv3346c and Rv355c genes located 26.3 kb apart (22). This LAM variant, called MTB RD^Rio^ is associated with multidrug resistance and distributed worldwide (22, 24–27). The prevalence of MTB RD^Rio^ varies in Latin America: 30 to 51.9% in Brazil (22, 28), 69.8% in Venezuela (19), 37% in Colombia (17, 21, 29), 31% in Peru (21, 30), 20% in Argentina (21), 10% in Paraguay (18) and 3% in Chile (21). However, there are no reports of the RD^Rio^ in Ecuador.

The aim of this study was to address the prevalence, drug resistance profile and population structure the MTB RD^Rio^ strains circulating in Ecuador.

## Materials and methods

### *Mycobacterium tuberculosis* isolates

**Figure 1:**
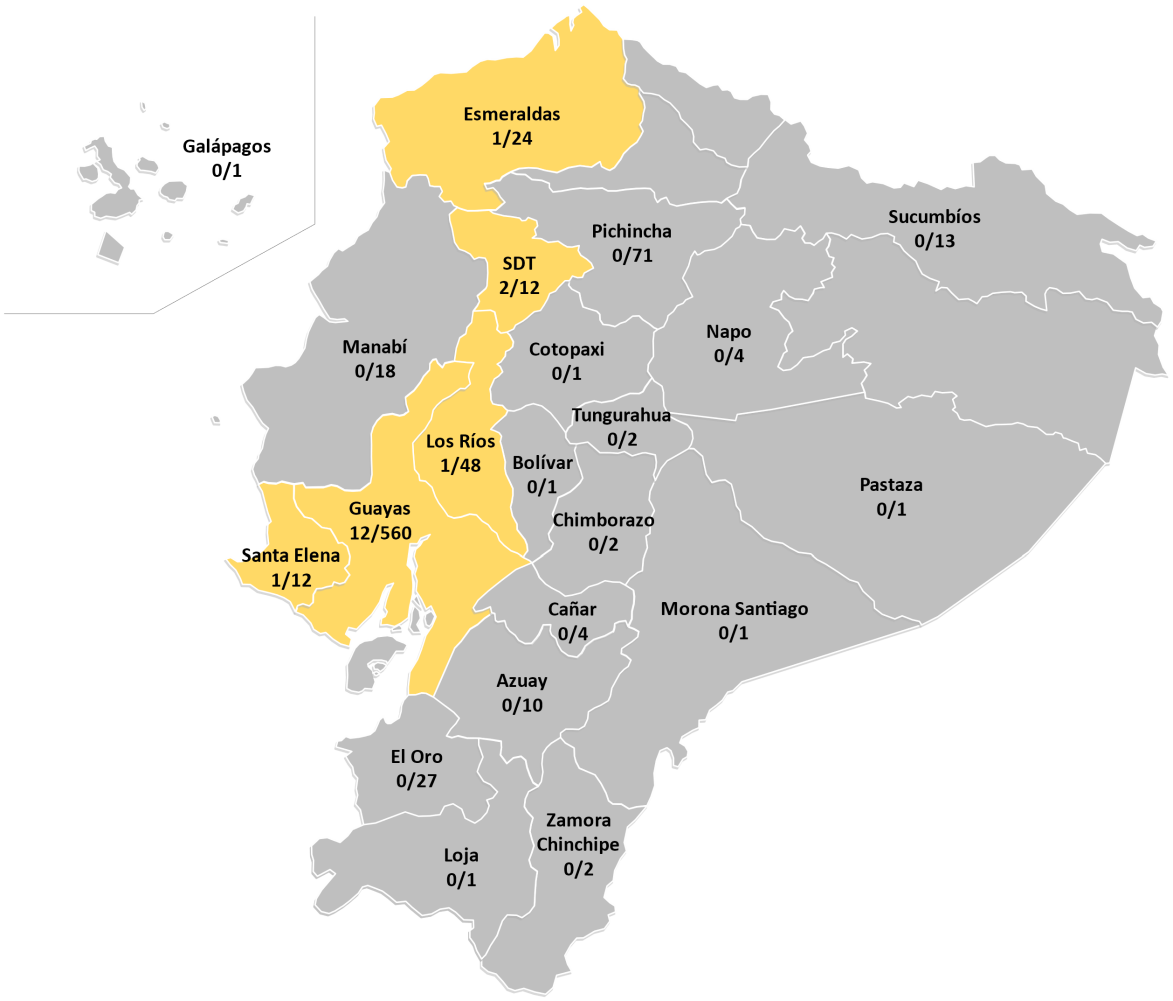
Ratio of RD^Rio^ isolates in each province of Ecuador (Total of RD^Rio^ isolates = 17). SDT, Santo Domingo de los Tsáchilas.

A collection of 814 *M. tuberculosis* isolates from years 2012 to 2016 stored at “Centro Nacional de Referencia para Micobacterias” from “Instituto Nacional de Salud Pública e Investigación Leopoldo Izquieta Pérez” in Guayaquil (Ecuador) were included in the study (Figure 1). MTBC isolates are routinely processed at INSPI laboratories, where culture and antibiotic resistance profiling for first-and second-line drugs used in *M. tuberculosis* therapy is performed for MTBC cultures following Pan American Health Organization guidelines (31–33). The samples were previously inactivated and stored for research purposes following the guidelines from this government center. The access to this MTB strains collection was approved by IRBs from “Instituto Nacional de Salud Pública e Investigación Leopoldo Izquieta Pérez” and University San Francisco de Quito (code 2017-023IN), both certified by Ministry of Public Health from Ecuador following guidelines from Declaration of Helsinki. All samples were anonymized, and no data of the patients were made available.

### *Mycobacterium tuberculosis* heat inactivation and DNA isolation

A small sample from cultures of MTBC isolates was collected and resuspended in TE buffer (10 mM Tris-HCl, 1 mM EDTA, pH 8.0), then inactivated by boiling at 95°C for 45 min. After this process, samples were centrifuged for 5 min at 10,000 g and the supernatant was directly used for genotyping, as reported elsewhere (34, 35).

### PCR screening for MTB RD^Rio^

A multiplex PCR developed for the detection of the RD^Rio^ pattern was performed using a two set of primers (Supplementary Figure S1): (1) a set of primers that target the IS1561’ fragment, located inside the region between the genes Rv3346c and Rv3355c, and used as a marker for wild type *M. tuberculosis* (530 bp); (2) another set of primers is used to flank the RD^Rio^ locus and bridge the deletion in RD^Rio^ strains (1175 bp) (36). The PCR reaction was prepared as follows: 12.5 μL of GoTaq^®^ Green Mastermix (Promega, Wisconsin, United States) (1X), 0.5 μL of each primer (0.2 μM), 5 μL of mycobacterial DNA and completed with Nuclease-Free water to a final volume of 25 μL. The multiplex PCR program was established as follows: initial denaturation at 94°C for 3 min, 35 cycles at 94°C for 30 s, 60°C for 30 s, 72°C for 1 min 30 s, and a final extension step of 72°C for 10 min. PCR products were run in 1.5%UltraPure^™^ Agarose (Invitrogen, California, United States) gels of 15 cm × 10 cm in 0.5X Tris-boric acid-EDTA (TBE) buffer at 100 V for 2 h using a ladder 100 bp Plus Opti-DNA Marker (Cat. No.: G016, Applied Biological Materials Inc., British Columbia, Canada) for size determination (22, 36).

### MTBC strains MIRU-VNTR genotyping

The method is PCR-based and allows the detection of different Mycobacterial Interspersed Repetitive Units (MIRU) located at multiple loci in the MTBC genome. Each MIRU allele is identified by a number; thus generating a numerical profile which is used for genotyping studies (35, 37). The PCR reaction was prepared individually to a final volume of 15 μL: 7.5 μL of GoTaq^®^ Green Mastermix (Promega, Wisconsin, United States) (1X), 0.75 μL of MIRU primers (0.5 μM) (38), 1 μL of mycobacterial DNA and completed with Nuclease-Free water. The following PCR program was established for all MIRUs: initial denaturation at 94°C for 5 min, 35 cycles at 94°C for 1 min, 54°C for 1 min 30 s, 72°C for 1 min 30 s, and a final extension step of 72°C for 10 min. Amplicons were run in 2% UltraPure^™^ Agarose (Invitrogen, California, United States) gels of 15 cm × 10 cm in 0.5X Tris-boric acid-EDTA (TBE) buffer at 100 V for 3 h using a ladder 100 bp Plus Opti-DNA Marker (Cat. No.: G016, Applied Biological Materials Inc., British Columbia, Canada) for size determination. MIRU allele identification was performed according to Supply et al. (38).

Results obtained were analyzed using the MIRU-VNTRplus web application^1^ (39). Lineage designation was performed by similarity search using 24-loci MIRU-VNTR data (Supplementary Data 1) and calculation of Minimum Spanning Tree (MST) and Neighbour-joining Tree (NJT) was performed to determine the population structure of Ecuadorian *M. tuberculosis* isolates.

### MTBC strains spoligotyping

The method is based on the presence/absence of 43 DNA spacer sequences, which are interspersed between 36 conserved loci located in the Direct Repeat (DR) region (37, 40). Spoligotyping was performed according to Kamerbeek et al. (41): PCR amplification of DR loci was performed using DRa and DRb primers and products were biotinylated and hybridized to a membrane containing oligonucleotides for each spacer sequence. After hybridization, the membrane is washed and then incubated in diluted streptavidin-peroxidase conjugate (Roche, United States). Membrane was exposed to chemiluminescent Amersham ECL reagents (GE Healthcare, United Kingdom) and located in an X-Ray cassette on a Hyperfilm ECL (Merck, United States). After the reaction, the film was inserted into a film developer solution in a dark room after which it is moved to the fixer solution. Thereafter, the film is dried and ready for the interpretation of the result. After developing the film a positive/negative signal is recorded in binary or octal formats for genotyping interpretations (Supplementary Data 1). The results are compared against the Fourth International Spoligotyping Database (SpolDB4) (42).

## Results

### Prevalence of MTB RDRio isolates in Ecuador

**Figure 2:**
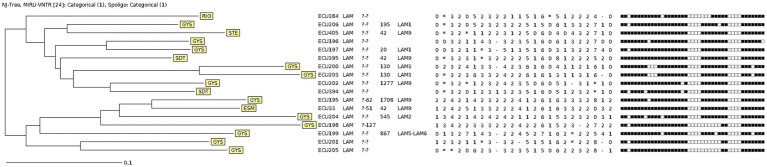
Neighbour-joining Tree of Ecuadorian MTB RD^Rio^ isolates using 24-loci MIRU-VNTR and Spoligotyping data. Labels use three-letter code to identify province of origin: ESM, Esmeraldas; GYS, Guayas; RIO, Los Ríos; SDT, Santo Domingo de los Tsáchilas; STE, Santa Elena.

A total of 814 *M. tuberculosis* isolates were analyzed by multiplex PCR for MTB RD^Rio^ identification (Figure 1). Of those, 17 strains generated a band pattern in the electrophoresis gel compatible with MTB RD^Rio^ (Figure 1; Table 1; Supplementary Figure S1), representing a 2.09% of the total MTBC population analyzed. Those MTB RD^Rio^ strains were assigned to LAM MTB subfamily by MIRU-VNTR (Table 1; Figure 2; Supplementary Data 1). Those 17 isolates were subsequently analyzed by spoligotyping, with 6 strains with undetermined lineage and 11 of them associated to LAM sublineage: five belong to LAM9, one to LAM5-LAM6, two belong to LAM3, one to LAM2 and two were identified as LAM1, according to the SpolDB4 (Table 1; Figure 2; Supplementary Data 1).

**Table 1:** MTB RD^Rio^ isolates in Ecuador.

ID	Procedence^*^	MIRU-VNTR	SITVIT	SpolDB4
ECU33	ESM	LAM	42	LAM9
ECU195	GYS	LAM	1708	LAM9
ECU196	GYS	LAM	–	–
ECU197	GYS	LAM	20	LAM1
ECU198	GYS	LAM	–	–
ECU199	GYS	LAM	867	LAM5-LAM6
ECU200	GYS	LAM	130	LAM3
ECU201	GYS	LAM	–	–
ECU202	GYS	LAM	1277	LAM9
ECU203	GYS	LAM	130	LAM3
ECU204	GYS	LAM	545	LAM2
ECU205	GYS	LAM	–	–
ECU206	GYS	LAM	195	LAM1
ECU384	RIO	LAM	–	–
ECU394	SDT	LAM	–	–
ECU395	SDT	LAM	42	LAM9
ECU405	STE	LAM	42	LAM9

*ESM, Esmeraldas; GYS, Guayas; RIO, Los Ríos; SDT, Santo Domingo de los Tsáchilas; STE, Santa Elena.

Regarding the geographic location of the 17 MTB RD^Rio^ strains, all of them were isolated from provinces in the Coastal Region of Ecuador: Guayas, Los Rios, Santo Domingo and Santa Elena (Figure 1).

### Drug resistance profile of MTB RD^Rio^ isolates from Ecuador

Drug susceptibility testing of MTB RD^Rio^ isolates was performed for first line antibiotics: 8/17 were resistant to isoniazid (47.1%), 1/17 was resistant to streptomycin (5.9%), 6/17 were resistant to rifampicin (35.3%) and no samples showed resistance to ethambutol and pyrazinamide; 9/17 isolates were sensitive to all first-line antibiotics (52.9%). Finally, 5/11 (29.4%) isolates were resistant to isoniazid and rifampicin, that means multi drug resistance (MDR) strains (Table 2). For the whole collection of 814 MTB strains included in the study, the MDR strains represented 10.9% (Table 3).

**Table 2:** Frequency of MTB RD^Rio^ isolates resistant to first-line therapy drugs and prevalence.

Drug Resistance Profile	Frequency	Prevalence (%)
Isoniazid resistant	8	47.1
Streptomycin resistant	1	5.9
Rifampicin resistant	6	35.3
Isoniazid + Rifampicin resistant (MDR)	5	29.4
Sensible to all tested drugs	9	52.9
TOTAL	17	

**Table 3:** Frequency of Ecuadorian MTB isolates resistant to first-line therapy drugs and prevalence.

Drug Resistance Profile	Frequency	Prevalence (%)
Isoniazid resistant	165	20.3
Streptomycin resistant	62	7.6
Rifampicin resistant	102	12.5
Ethambutol resistant	9	1.1
Pyrazinamide resistant	19	2.3
Isoniazid + Rifampicin resistant (MDR)	89	10.9
Sensible to all tested drugs	455	55.9
Without information	159	19.5
TOTAL	814	

### Phylogenetic analysis of MTB RD^Rio^ isolates from Ecuador

The population structure of the MTB RD^Rio^ isolates was determined through the analysis of 24-loci MIRU-VNTR and spoligotyping using the MIRU-VNTRplus platform (Supplementary Data 1), as it is detailed in the Neighbour-Joining Tree (NJT) presented in Figure 2. Although several spoligotype patters were repeated for 2 or 3 MTB RD^Rio^ strains, none of the strains shared the same MIRU-VNTR pattern. Based on total identity in 24 loci for MIRU-VNTR, no actively transmitted MTB RD^Rio^ clones were found (Figure 2).

**Figure 3:**
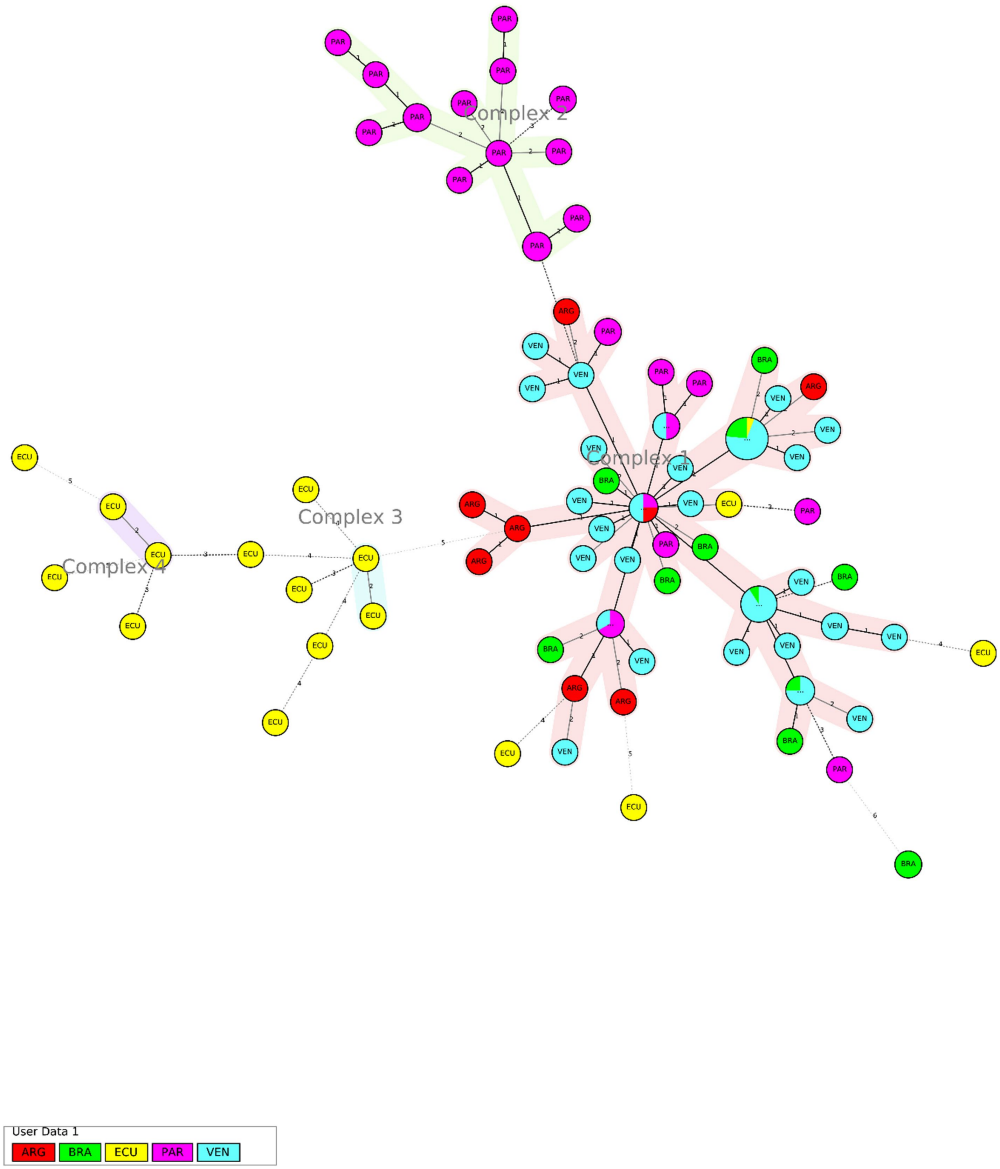
Minimum Spanning Tree of Latin American RD Rio isolates isolates using 12-loci MIRU-VNTR data. Maximum locus difference within a clonal complex is 2. Labels use three-letter code to identify country of origin (ARG, Argentina; BRA, Brazil; ECU, Ecuador; PAR, Paraguay; VEN, Venezuela).

Additionally, a phylogenetic analysis was done using 12-loci MIRU-VNTR, including all the MTB RD^Rio^ strains reported in the bibliography (18, 43), as well as the strains identified in the current study for Ecuador. This analysis is detailed in the Minimum Spanning Tree (MST) in the Figure 3, where the maximum locus difference within a clonal complex was set at 2. MST included 17 MTB RD^Rio^ isolates from Ecuador, 8 from Argentina, 31 from Paraguay, 51 from Venezuela and 15 from Brazil. Four clonal complexes of MTB RD^Rio^ isolates are well defined in the MST: two clonal complexes comprising isolates exclusively from Ecuador, one clonal complex comprising isolates exclusively from Paraguay, and one large clonal complex including MTB RD^Rio^ isolates from Ecuador, Argentina, Brazil, Paraguay and Venezuela. Interestingly, 12 of 17 MTB RD^Rio^ strains from Ecuador are well differentiated from strains from the other South American countries.

## Discussion

We found the lowest prevalence of MTB RD^Rio^ subfamily described for South America in Ecuador (2.09%), while neighbor countries like Peru and Colombia have an MTB RD^Rio^ prevalence over 30% (21). Although some biological advantages that improve virulence and transmissibility over other genotypes has been described for MTB RD^Rio^ (36, 44, 45), this could explain its successful transmission in some countries like Venezuela, Brazil, Peru and Colombia, but not in Ecuador (22, 46, 47). In fact, two recent studies have shown a very limited transnational transmission of MTB between Ecuador, Colombia and Peru that would also explain the low prevalence of MTB RD^Rio^ sublineage found in our study (48, 49).

The phylogenetic analysis of MTBC RD^Rio^ strains identified in Ecuador suggests a regional cluster associated to provinces in the Coastal Region but with an elevated genetic diversity and lack of active transmission chains. While a single large clonal complex includes MTB RD^Rio^ isolates from all South American countries was found, 70% of RD^Rio^ strains from Ecuador were clearly segregated in two clonal complexes. Additionally, the high genetic diversity of MTB RD^Rio^ strains in Ecuador was also confirmed by the multiple patterns of LAM spoligotypes found in our study.

The RD^Rio^ strains from Ecuador were found strongly associated to MDR TB in our study. The reported value of 29.4% MDR strains is clearly over either the 9% of MDR TB estimated for Ecuador for 2017 (6) or the 10.9% of MDR TB strains in the collection included in this study. This result points out that MTB RD^Rio^ strains should be considered of concern due to their potential to mutate to drug resistance phenotypes (21, 28, 50, 51).

Our study has two main limitations. First, we could only have access to 814 MTB strains from the national reference located in Guayaquil, so geographic bias in this collection could happened with over representation of provinces closer to this city. Second, our study was done with samples from years 2012–2016, prior to the massive Venezuelan migration that arrived to Ecuador since 2018 following this country economic collapse (52, 53). Considering that the highest prevalence of MTB RD^Rio^ in South America has been described for Venezuela (19), the current scenario for MTB RD^Rio^ prevalence in Ecuador could have changed due to importation of cases and further studies with MTB strains collection from recent years are needed.

In conclusion, we report for the first time the presence of MTB RD^Rio^ in Ecuador and its strong association to MDR-TB. We encourage the national surveillance program in Ecuador to follow up the potential expansion of this MTB strains of concern considering the TB burden associated to COVID-19 pandemic and the recent massive regional patters of migration from Venezuela where MTB RD^Rio^ is highly present.

## Data availability statement

The original contributions presented in the study are included in the article/Supplementary material, further inquiries can be directed to the corresponding author.

## Author contributions

BC-R: Conceptualization, Formal analysis, Investigation, Methodology, Writing – original draft, Writing – review & editing. GF-S: Conceptualization, Data curation, Investigation, Methodology, Resources, Supervision, Writing – review & editing. JB-M: Formal analysis, Methodology, Validation, Writing – review & editing. GC-F: Data curation, Formal analysis, Investigation, Methodology, Writing – review & editing. NJ-P: Data curation, Formal analysis, Methodology, Writing – review & editing. CC-F: Data curation, Formal analysis, Validation, Writing – review & editing. JA-M: Project administration, Resources, Supervision, Writing – review & editing. JH: Funding acquisition, Methodology, Resources, Supervision, Validation, Writing – review & editing. MG-B: Conceptualization, Data curation, Formal analysis, Funding acquisition, Investigation, Methodology, Project administration, Resources, Supervision, Writing – original draft. SO: Methodology, Resources, Project administration, Writing- review & editing.

## Footnotes

^1^www.miru-vntrplus.org
