# Supplementary material for: Prevalence, drug resistance, and genotypic diversity of the RDRio subfamily of Mycobacterium tuberculosis in Ecuador: a retrospective analysis for years 2012–2016
Source: Front Public Health. 2024 Apr 4;12:1337357. doi: 10.3389/fpubh.2024.1337357 (PMC11060180; doi:10.3389/fpubh.2024.1337357)
Supplement: Supplementary file 2 [file Image_1.pdf]

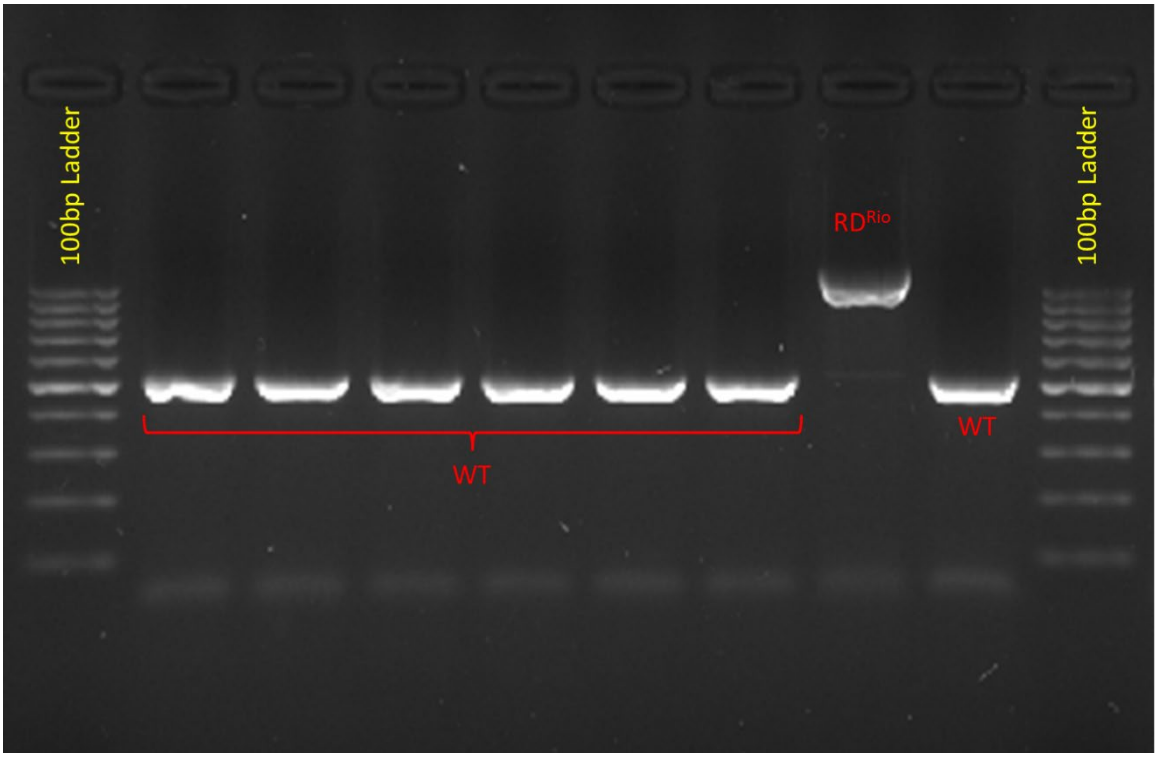

Supplementary Figure 1. RD^{Rio} and wild type (WT) amplicons after multiplex PCR for RD^{Rio} screening, as observed in 1.5% agarose gels. The PCR reaction is based in the use of one set of primers that target the IS1561′ fragment located inside the region between the genes Rv3346c and Rv3355c, thus identifying wild type *M. tuberculosis* by the presence of an amplicon of 530bp. A deletion occurs by homologous recombination in RD^{Rio} strains that excises all elements between Rv3346c and Rv3355c genes, including the IS1561′ fragment, and produces a new fusion gene designated as Rv3346c/55c. Another set of primers is used to flank the deletion (RD^{Rio} locus) and amplify a fragment in Rv3346c/55c of 1175bp.
